# Magnetic Resonance Spectroscopy for Cervical Cancer: Review and Potential Prognostic Applications

**DOI:** 10.3390/cancers16112141

**Published:** 2024-06-05

**Authors:** Zohaib Iqbal, Kevin Albuquerque, Kimberly L. Chan

**Affiliations:** 1 Department of Radiation Oncology, UT Southwestern Medical Center, Dallas, TX 75235, USA; kevin.albuquerque@utsouthwestern.edu; 2 Advanced Imaging Research Center, UT Southwestern Medical Center, Dallas, TX 75235, USA; kimberly.chan@utsouthwestern.edu

**Keywords:** MRS, NMR, cervical cancer, choline, fatty acids, lipids, biomarkers

## Abstract

**Simple Summary:**

Cervical cancer is the fourth most prominent cancer in women worldwide. Early cancer detection, timely treatment, and prognostic marker identification are vital to ensure that patients have improved outcomes. Magnetic resonance spectroscopy (MRS) is a powerful tool for detecting metabolites in vivo. This review article covers the role of MRS for cervical cancer for diagnosis, treatment response evaluation, and future applications of this technology.

**Abstract:**

This review article investigates the utilization of MRS in the setting of cervical cancer. A variety of different techniques have been used in this space including single-voxel techniques such as point-resolved spectroscopy (PRESS) and stimulated echo acquisition mode spectroscopy (STEAM). Furthermore, the experimental parameters for these acquisitions including field strength, repetition times (TR), and echo times (TE) vary greatly. This study critically examines eleven MRS studies that focus on cervical cancer. Out of the eleven studies, ten studies utilized PRESS acquisition, while the remaining study used STEAM acquisition. These studies generally showed that the choline signal is altered in cervical cancer (4/11 studies), the lipid signal is generally increased in cervical cancer or the lipid distribution is changed (5/11 studies), and that diffusion-weighted imaging (DWI) can quantitatively detect lower apparent diffusion coefficient (ADC) values in cervical cancer (2/11 studies). Two studies also investigated the role of MRS for monitoring treatment response and demonstrated mixed results regarding choline signal, and one of these studies showed increased lipid signal for non-responders. There are several new MRS technologies that have yet to be implemented for cervical cancer including advanced spectroscopic imaging and artificial intelligence, and those technologies are also discussed in the article.

## 1. Introduction to MRS

In vivo magnetic resonance spectroscopy (MRS) [1] and MRS imaging (MRSI) [2] are non-invasive methods to measure biochemicals in the body. Both methods are used widely in biomedical research to study the metabolism of living systems. Although MRS and MRSI are also used in clinical practice, they have yet to be widely adopted. Regardless, MRS is a highly researched field with continual technological advances that show promise in moving MRS/MRSI to be adopted by general medical practice [3].

Because of its relatively low sensitivity, only small, mobile molecules present in millimolar concentrations can be detected [4]. In addition, most studies have generally focused on the proton (^1^H) nucleus due to its naturally large abundance in the human body and its high gyromagnetic ratio, which results in a higher detection sensitivity. A downside to ^1^H-MRS, however, is its lack of spectral dispersion, which is exacerbated at lower field strengths [5]. As such, clinical MR scanners, which typically have low-to-medium field strengths of 1.5 T or 3 T, have a relatively limited spectral resolution that restricts the number of metabolites that can be reliably measured. Regardless, these scanners can still detect a large range of metabolites including choline and phosphocholine, membrane-linked metabolites, and lipids [6]. Metabolites detectable in the cervix that likely play a role in cervical cancer metabolism will be further discussed in Section 2.

### 1.1. Localization Methods

Due to its high signal-to-noise and robustness to experimental imperfections (relative to multi-voxel localization methods), single-voxel localization methods [7,8] are most commonly used. Nearly all single-voxel localization methods use three slice-selective radiofrequency pulses along orthogonal planes to detect a signal from a select region where the pulses intersect. Voxel sizes typically range from 4 to 9 cm^3^ depending on the targeted region, technique and field strength used, and metabolite(s)-of-interest.

**Figure 1:**
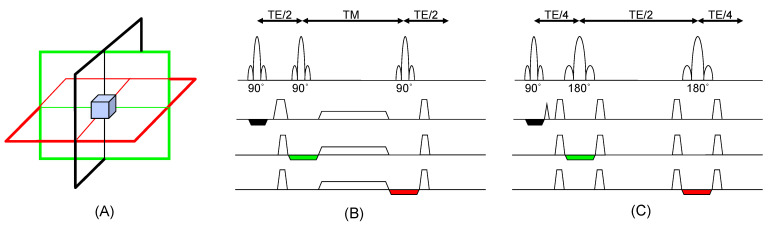
(**A**) Single voxel localization is achieved by acquiring data from the intersection of slice-selective pulses applied in orthogonal directions (black, green, and red) (**B**) The STEAM localization technique is shown, which utilizes three 90° pulses. (**C**) The PRESS localization technique is displayed, which utilizes a 90° excitation pulse followed by two 180° pulses. In the figure, TE is the echo time and TM is the mixing time, and these values can range from a few milliseconds to hundreds of milliseconds. STEAM forms a stimulated echo, whereas PRESS forms a spin echo, which can result in different T2 weighting of the spectra. The black, green, and red can be iterated during an experiment to yield MR spectroscopic imaging.

STimulated Echo Acquisition Mode (STEAM) [9] and Point RESolved Spectroscopy (PRESS) [1] are the two most common types of single-voxel MRS acquisition techniques. Figure 1 depicts the STEAM (Figure 1B) and PRESS (Figure 1C) sequences. Both yield spectra that can be analyzed both qualitatively and quantitatively, however, there are a few practical differences between the two techniques. STEAM is generally preferred at higher magnetic fields such as 7 T [10] due to radiofrequency (RF) power requirements. PRESS generally offers more signal-to-noise and the spectra are T2-weighted, rather than T2*-weighted in STEAM, so it is preferred at lower field strengths and has been used in a variety of clinical applications [11].

While single-voxel methods have been traditionally used, it has a major limitation of being spatially-limited. As such, multi-voxel techniques such as MRSI [12] are needed if there are multiple regions of interest, the affected region is unknown, or there is significant heterogeneity across a region of interest, as is common in tumors. Unlike MR imaging (MRI), frequency encoding cannot be performed as the gradient played during readout would scramble the chemical shift encoding, which is difficult to disentangle. Therefore, MRSI is typically performed by adding phase-encoding gradients to the aforementioned localization methods. In most cervical cancer studies performed to date at clinical field strengths, these MRSI acquisitions have been most commonly performed with PRESS localization.

### 1.2. Applications in Medicine

Due to the rich chemical information provided non-invasively without ionizing radiation, MRS has been applied to study a variety of different diseases. Most of these have been in the brain and have included the study of neurodegenerative diseases [13,14], psychiatric disorders [15,16], and brain tumors [10,17,18]. However, MRS is increasingly being used to study diseases and disorders affecting a range of other bodily organs including spinal cord injury [19], myocardial infarction [20], peripheral arterial disease [21], prostate cancer [22,23], and cervical cancer [24,25]. MRS has also been applied to study a range of metabolic disorders including diabetes [26] and liver disease [27]. In prostate cancer, MRS has been used to identify target subvolumes and may also play a role in predicting tumor response and outcomes [28]. It has been shown that MRI/MRS can be used to design simultaneous integrated boost (SIB) plans for prostate radiotherapy, where the dominant intraprostatic lesion also receives more dose than the prostate [29]. Although cervical cancer has been less well-studied than prostate cancer, several studies have used MRS to help understand, diagnose, and assess treatment responses in patients with cervical cancer.

## 2. Background on Metabolic Pathways of Interest in Cervical Cancer MRS

It is well-known that cancer differs from healthy cell metabolism, and that signaling pathways are also greatly altered in cervical cancer [30,31,32,33]. One specific example of metabolism change is the Warburg effect [34], which describes the change in the preferred glucose metabolic pathway and is a well-understood phenomenon. In healthy cells, glucose undergoes glycolysis to produce pyruvate. If oxygen is available, pyruvate will go through the oxidative phosphorylation process, which is energy efficient. However, if oxygen is unavailable, the pyruvate will be fermented to produce lactate, which produces much less energy. In cancer cells, fermentation is prevalent, even in environments abundant with oxygen, drastically altering lactic acid build-up and pyruvate by-products. Cervical cancer MRS, however, has predominantly focused on two metabolic pathways: choline and fatty acids.

### 2.1. Metabolic Pathways of Choline in Cancer

Choline is a well-studied metabolite in several pathologies, and the molecular pathways of this compound have been studied in cancer extensively. Choline is prominent in different forms in the body, typically in the forms of free choline (Cho), phosphocholine (PCh), and glycerophosphocholine (GPC). Changes in these choline species are indicative of alterations in the enzymes that produce or utilize choline. This includes key enzymes such as choline kinase, phospholipase C, lysophospholipase, and others [35,36,37]. There are also important choline transporter enzymes that transport free choline from the extracellular space to the intracellular space, which may be affected [36,37].

In the in vivo ^1^H MRS literature, it is well-reported that the concentration and distribution of these choline compounds are modified in cancerous tissues [38]. Notably, a significant elevation of total choline (tCho), a summation of Cho, PCh, and GPC signals, has been consistently reported to be elevated in various cancer pathologies [3]. In particular, several forms of brain cancer have demonstrated significant elevations in tCho including meningiomas [39] and glioblastomas (GBMs), and this elevated signal may be useful in diagnosis or targeted therapies. In fact, there is an ongoing clinical trial escalating the radiation dose to the regions of the tumor with elevated choline and decreased N-acetyl aspartate (NAA) with promising preliminary results that indicate that the volume of spectroscopically abnormal tissue is a better biomarker of progression-free and overall survival than the volume of residual post-contrast enhancement [40]. Prostate cancer and breast cancer have also demonstrated increased tCho levels. Furthermore, breast cancer has also demonstrated a redistribution of GPC into PC [41] associated with malignancy. Choline may also play an important role in cervical cancer as a potential biomarker holding both diagnostic and prognostic value. Studies investigating choline in cervical cancer and other gynecological cancers are described in Section 3.

### 2.2. Metabolic Pathways of Lipids in Cancer

Fatty acid metabolism also plays a large role in the energy metabolism of cancer [42]. Cellular membrane and signaling molecules typically rely on fatty acids or lipids. One example is the use of lipids in the phospholipid bilayer, which encases cells and acts as a barrier between the internal organelles and the external environment. In cancer cells, the proliferation of cells is supported by the abundance of lipids in order to create these membranes. Lipids also play a vital role in the storage of energy such as the storage of triglycerides in fat cells, which can be released as an energy source at a later time. In the case of cancer cells, these stored fatty acids can be released to act as building blocks for membranes. While the exact role that altered lipid storage plays in cancer is still not well-understood, it is well-known that an abundance of lipids will support increased proliferation. Therefore, fatty acid metabolism and synthase may act as therapeutic targets in the case of cervical cancer [43,44]. Several studies have detected lipids in cancer by employing ^1^H MRS, most notably in the cases of breast [45,46] and brain cancer [47,48]. Although this space has not been as thoroughly explored as choline metabolism, there are several studies that have evaluated lipid levels in cervical cancer, as discussed below.

## 3. In Vivo MRS Studies in Cervical Cancer

Early cervical cancer MRS studies have focused on diagnosing the disease and identifying the key metabolites of interest. One of the earlier studies for ^1^H-MRS of cervical carcinoma was performed in 1998 by Lee et al. [49]. In this study, fifty-one patients were included, forty-four of which were pathologically confirmed squamous cell carcinoma and seven of which were confirmed as adenocarcinoma. Spectra were acquired on a 1.5 T scanner using a body transmit coil and an endovaginal surface receive coil, which was placed in the posterior fornix of the vagina by a gynecologist. A PRESS sequence was utilized with TR = 3000 ms and TE = 20 and 135 ms, and the voxel was placed inside apparent cancerous tissue based on the T2-weighted images, or in the center of the cervix itself if abnormal tissue was not visible. The voxel size was between 1 and 3 cc. The key findings of the study were that squamous cell carcinoma could be discriminated from the normal cervix by using the triglyceride peak at 1.3 ppm (89% sensitivity, 57% specificity, 84% accuracy), and adenocarcinoma could be discriminated from the normal cervix by using the lipid peak at 2.0 ppm (86% sensitivity, 100% specificity, 98% accuracy), potentially offering an alternative to biopsy. While the results were promising, the qualitative nature of the analysis, which focused on the simple presence of peaks rather than the concentration values, was one weakness of the study. A more quantitative and statistical approach is necessary to demonstrate true cancer sub-type discrimination.

Studies looking into the role of choline metabolism in cervical cancer have also showed interesting results. One study, conducted by Booth et al. [50], reported the results of in vivo MRS acquired on a variety of gynecological tumors. The study included fourteen patients with ovarian cancer, eleven patients with cervical cancer, and four patients with uterine cancer. The spectra were acquired on a 3 T MR scanner with a torso coil for acquisition. The voxel position and size was dependent on the tumor location, ranging from 5.3 to 81.3 mm^3^, and the PRESS sequence was used with the following acquisition parameters: TR/TE = 1500/72 ms and 128 water-suppressed excitations. Choline peaks were prominent in 93%, 73%, and 100% of ovarian, cervical, and uterine cancers, respectively. However, no significant differences were found in the choline signals based on the level of malignancy of the tumor specified as the tumor stage. This study may have had more definitive results with a larger sample size and a more statistical approach for comparing data.

Another study by De Silva et al. [51] also demonstrated interesting results regarding choline. The group carried out an in vivo spectroscopic imaging study on forty-seven women, nineteen of which had cervical intraepithelial neoplasia (CIN) and twenty-eight of which had cervical cancer. The MRSI study was performed on a 1.5 T scanner using an endovaginal ring coil and PRESS voxel localization. The spectroscopic imaging was localized in the coronal plane, with a 16 × 16 voxel acquisition, slice thickness of 15 mm, FOV of 120 × 120 mm^2^, TR/TE = 888/135 ms, and four signal averages. The epithelial and stromal voxels were identified, and metabolic signals from choline and lipids were quantified from these separate regions. The results showed that patients with cervical cancer had significantly elevated choline values (*p* = 0.033) compared to patients with CIN in the epithelium, however, there were no significant differences in the stroma. Additionally, no differences in any of the triglycerides or other lipids were found.

Lipid peaks have also been shown to undergo alterations in cervical cancer. Mahon et al. [52] analyzed MRS data acquired from eleven controls and twenty-seven patients with cervical cancer. Importantly, their work focused on comparing in vivo results to ex vivo magic angle spinning (MAS) MR spectroscopy. The in vivo MR spectra were acquired on a 1.5 T scanner using an endovaginal coil. A 3.6 cc voxel was placed either on healthy tissue or on cancerous tissue, with the voxel containing at least 50% abnormal tissue as seen on a T2-weighted MR image. A PRESS sequence was used for the acquisition with TR/TE = 1600/135 ms and a total of 128 signal averages. The MAS data were acquired on tissue samples taken from the biopsy of six healthy controls (biopsies were taken unrelated to cancer) and nineteen of the cervical cancer patients. An 11.75 T machine was used to acquire the MAS spectra, which had a much higher signal-to-noise ratio than the in vivo MRS data. Overall, the in vivo results demonstrated the presence of choline and elevated lipid species in the cervical cancer MRS using a more qualitative approach. In addition, the MAS spectra demonstrated significant quantitative differences in the -CH_2_ and -CH_3_ lipid species as well as choline. Another report from Mahon et al. [53], utilizing a similar approach to the above, focused on the relationship between choline, lipids, and tumor load. The in vivo quantitative results showed significant increases in the lipid signal for the cervical cancer MRS (*p* < 0.05). However, there was no correlation between the tumor load and metabolite levels.

A study by Lin et al. [54] looked into the potential value of MR spectroscopy for the prediction of poor prognostic human papillomavirus (HPV) genotypes. The group acquired spectra from fifty-two cervical cancer patients on a 3 T system using an external spine coil and body coil. The spectra were acquired using a PRESS sequence with TR/TE = 2000/35 ms and 128 averages. The spectra were analyzed using the LCModel (version 6.3-0 K) software [55] following eddy current correction. LCModel is a peak fitting software that is able to incorporate prior knowledge of signals to fit spectra in a linear least-squares manner, and has been shown to provide superior quantification relative to simple spectral peak maximums or peak integrals for quantification, as performed in many earlier studies. In this study, the lipid peaks resonating at 0.9, 1.3, and 2.0 ppm and the total choline peak at 3.2 ppm were quantified. The results demonstrated that the 0.9 ppm lipid peak signal was significantly increased (*p* = 0.032) for patients with poor prognostic HPV genotypes, defined as HPV18, 39, 45, and the absence of HPV infection. Furthermore, the elevated lipid peak could be used to discriminate between HPV18 and HPV16 with a sensitivity of 83%, a specificity of 90%, and an area under of the curve (AUC) of 0.82 when analyzing the receiver operating characteristic (ROC) curve. No significant differences in choline values were reported.

Lipid signals in cervical cancer have also been investigated at high field strengths. Arteaga de Castro et al. [25] were the first group to acquire 7 T spectra of cervical cancer. In their study, they included ten women with Stage IB1–IIB2 cervical cancer at a point after definitive therapy was already initiated. All MRS data were acquired on a 7 T system. The coils used for the acquisition were a pelvis coil and an endorectal coil. For the 7 T acquisition, a STEAM sequence was used for both single-voxel and CSI acquisitions with VAPOR for water suppression. The single-voxel parameters included: TR = 1400 ms, TE = 36–75 ms, 192 averages, and voxel sizes varying from 20 to 50 mm^3^. The CSI scan had a TR/TE = 1400/10 ms and a 5 × 5 × 5 mm^3^ voxel size covering a 30 × 30 mm^2^ FOV. The spectra were quantified using NMRWizard (version 2012-10-09), which is a LCModel based software. While no statistically significant results were found, the authors were able to take advantage of the higher field strength to distinguish fatty acid peaks that overlap at lower field strengths. Additionally, the 2.1 ppm/1.3 ppm ratio was higher as a function of tumor grade, demonstrating a trend of more unsaturated fatty acids in poorly differentiated tumors.

Treatment response has been monitored using MRS, and studies have focused on both choline and lipid signals. One study was undertaken by Allen et al. [56], which used MRS for initial detection and follow-up after the radiation treatment of cervical cancer. A total of eight healthy volunteers, sixteen pre-treatment patients, and eighteen post-treatment patients were scanned. All spectra were acquired on a 1.5 T scanner using a PRESS sequence with TR/TE = 1600/140 ms. To minimize invasiveness, an external pelvic surface coil was used for the acquisitions rather than an endovaginal coil. Prior to treatment, patients had elevated choline levels. For patients with no disease at time of follow-up, no elevated choline was detected, whereas for patients with local recurrence or biopsy confirm recurrence, the choline levels remained high. While this study demonstrates the non-invasive detection of treatment response using MRS, it does have a few weaknesses that need to be highlighted. The type of radiation treatment was not specified in the publication, and it is not clear whether the same patients were scanned pre-treatment and post-treatment. In addition, no quantification or statistical analysis was used in this study, thus limiting the results.

Another work focusing on therapy response was by Dolciami et al. [24], where they studied the role of MR spectroscopy as a prognostic test for assessing the response of cervical cancer to neoadjuvant chemotherapy. Seventeen patients were included in their study and these patients were scanned utilizing a 3 T scanner. A PRESS acquisition with TR = 1500 ms and TE = 28 and 144 ms with a voxel size of 18 × 18 × 18 mm^3^ was used. The shorter TE spectra were used for metabolite quantitation, which was performed using LCModel. The patients were split into good responders (GR), partial responders (PR), and non-responders (NR) following neoadjuvant chemotherapy based on the radiological response determined by two radiologists. Lipids at 1.28 ppm, tCho, and the ratio between the two (Lipids/tCho) were all significantly different between the GR and PR-or-NR groups. The lipids were significantly elevated in the PR-or-NR group (*p* = 0.04), whereas tCho was elevated in the GR group (*p* = 0.04). This finding is interesting because the tCho elevation in GR is in directly conflict with the results presented by Allen et.al. in their work, although the results could also be indicative of another parameter difference or condition. For example, the differences in TE employed by the two studies suggest that perhaps a difference in the T2 values could also play a role in these contrasting findings. T2 values in cervical cancer are unknown, and it is not clear how therapy may change these relaxation times. Similarly, T1 relaxation values may also differ between these types of acquisitions. A study with a larger sample size needs to be conducted in order to determine whether tCho is actually increasing for GR patients.

Multimodal imaging is a powerful tool in MRI. One study conducted by Rizzo et al. [57] evaluated the role of diffusion weighted imaging (DWI) in combination with spectroscopy as an early response marker to non-surgical therapies for cervical cancer. A total of sixteen patients underwent radiation therapy, chemotherapy, or chemo-radiation therapy and were stratified into responders and non-responders at the end of the therapies, and no evidence of disease (NED) or progression of disease (PD) after a five-year follow-up. The patients in the study had MRIs acquired on a 1.5 T scanner using an 18-channel external coil at pre-treatment, mid-treatment, and end of treatment timepoints. The MRS acquisition parameters were: voxel size = 3.8 cc, TR/TE = 2000/135 ms, and averages = 192. For responders, the absolute apparent diffusion coefficients (ADC) of the tumor increased (*p* = 0.0001) from the baseline to mid-treatment. Comparatively, the non-responders had no changes. When analyzing the percent increase compared to the baseline, however, there were no statistically significant changes at mid-treatment or at the end of treatment for the ADC differences. Similarly, tCho ratios with respect to water also demonstrated no significant changes throughout the course of treatment.

Another group that leveraged the capabilities of DWI was Payne et al. [58]. They conducted research to investigate the diffusion characteristics and metabolism of stage 1 cervical cancer. The research included sixty-two patients and used a similar approach and the same methodology described above in De Silva et al. [51] The diffusion sequence utilized five different b-values, 0, 100, 300, 500, and 800 s/mm^2^, and the authors employed in-house software (IDL 6.1) to calculate the ADC values from these data. The ADC values were significantly lower in well/moderately differentiated tumors and poorly differentiated tumors compared to normal tissue. There were no significant changes in tCho, and furthermore, there were no significant correlations found between ADC and tCho.

As displayed in Table 1, several parameters differed between the studies presented. For example, the TE of the acquisition plays a large role in the effects of T2 decay on the spectra, and shorter TEs will result in less T2 decay. Lipids will typically have a higher signal at these shorter TE values. The results of the studies, also summarized in Table 1, displayed consistent results for the most part. In general, lipid and tCho signals are higher in cervical cancer compared to controls. Lipids, and specifically the distribution of lipid species, may act as a biomarker for disease progression and response to therapy. tCho may play a leading role as a biomarker for diagnosis; however, it is not clear how well this biomarker may perform for treatment prognosis or response.

## 4. Future of MRS in Cervical Cancer

There have been major improvements in the field of MRS in recent decades. Most of the studies discussed above have not leveraged these recent advances, and therefore there may be significant potential to advance cervical cancer characterization by utilizing MRS. While none of the applications below have directly been applied to cervical cancer, there is a strong possibility that using the technologies may improve the signal, spatial resolution, or metabolite discrimination.

### 4.1. Technological Improvement in Acquisition

One of the aspects that was leveraged in a few recent studies, but could be utilized more prominently, was field strength [5]. The signal-to-noise ratio (SNR) for metabolites generally increases linearly as a function of field strength, meaning a 3 T acquisition will have roughly double the SNR compared to a 1.5 T acquisition. In addition to improved SNR, the spectral dispersion increases as a function of field strength, allowing for better peak identification.

A technology that was not incorporated as much in cervical cancer MRS studies was an improved localization technique. While PRESS and STEAM offer adequate localization, techniques such as LASER or semi-LASER acquisitions [59,60] exist, which have the potential to provide better signal localization and minimize the chemical shift displacement effects. This is especially important for higher field strengths of 7 T, where the sLASER adiabatic refocusing pulses result in reduced chemical shift displacement error and sensitivity to static and radiofrequency inhomogeneity. A downside to these types of techniques, however, is that they require longer repetition times to accommodate the increased specific absorption rates of the adiabatic pulses. However, the overall result is improved sensitivity, which can be significant when dealing with lower SNR signals. Shimming improvements such as the use of nonspherical harmonic shim hardware [61] are also possible, which would help further improve localization and SNR and allow for a better distinction of metabolites with neighboring resonances.

Spectral editing is another technology that has not been applied in cervical cancer MRS. This technique targets J-coupled metabolites and is capable of isolating their signal to enhance quantitation [62,63]. From the literature, it is clear that lipid metabolism may be of particular interest in cervical cancer prognosis, so using lipid editing to resolve in vivo unsaturated lipid resonances [64,65] may offer a unique mechanism to identify biomarkers.

### 4.2. Spectroscopic Imaging

MRSI has been severely under-utilized and was only used in two studies; furthermore, metabolic maps of the different biochemicals were not present in the literature. There have been significant advances in the field of MRSI, mostly facilitated by various acceleration methods including the shortening of repetition times [66], under-sampling of k-space [67,68,69], and spatial-spectral encoding [70]. Metabolic maps may allow for better tumor delineation, may serve a prognostic role, or may help guide therapy. Specifically, regions with abnormal metabolite signals may benefit from higher doses of radiation, similar to the approach described by Ramesh et al. [40] in the phase II clinical trial for GBM.

### 4.3. Artificial Intelligence

Machine learning and artificial intelligence (AI) is another emerging technology that has shown promise in MRS metabolite quantitation [71,72] as well as in the field of MRSI acquisition [73]. The major advantage of AI is that patterns in data can often be readily detected, offering another avenue for biochemical or radiomic identification. In general, AI has several applications in the space of medical imaging, particularly in MRI [74]. It is also possible to use machine learning techniques for cervical cancer prognosis [75], however, a large amount of MRS data would first need to be acquired in a standardized manner to accomplish this. With a plethora of data, it would be possible to build AI that can help guide tumor grading or diagnosis [76,77]. Furthermore, AI may be able to aide with planning a patient’s course of treatment [78], monitoring this treatment [79], and making adjustments as necessary based on biomarker changes. Combining MRS data with DWI and/or T2-weighted MRI information may ultimately allow for patient-tailored treatments and pave the road for personalized medicine.

## 5. Conclusions

Magnetic resonance spectroscopy offers a unique lens into understanding, diagnosing, and assessing treatment response for cervical cancer. Currently, choline and fatty acid metabolism have been found to be significantly different in cervical cancer patients compared to normal subjects. However, the studies presented in this review article had lower sample sizes and variable acquisition parameters and quantification methods, which makes it difficult to generalize the results. Thus, there is a need to conduct standardized MRS studies using modern acquisition techniques to determine the best use for MRS in the diagnostic and prognostic clinical setting for cervical cancer. A large standardized MRS dataset would enable the use of state-of-the-art technology such as artificial intelligence to improve cancer treatment and allow for personalized treatment. Therefore, efforts should be made to continue cervical cancer MRS studies.

## Figures and Tables

**Table 1:** Summary of cervical cancer MRS findings.

Number of Patients	Field Strength (T)	MRS Sequence	TR/TE (ms)	Clinical Finding	Reference
51	1.5	PRESS	3000/20 and 3000/135	Lipid peaks can be used to discriminate between squamous cell carcinoma and adenocarcinoma	[49]
14	3	PRESS	1500/72	tCho peaks prominent in different GYN cancers, however, cannot discriminate level of malignancy	[50]
47	1.5	PRESS CSI	888/135	tCho are elevated in cancer compared to cervical intraepithelial neoplasia (*p* = 0.033)	[51]
39	1.5	PRESS	1600/135	tCho and lipids are elevated in cervical cancer patients	[52]
27	1.5	PRESS	1600/135	Lipid peaks are significantly elevated in cervical cancer, however there was no correlation with tumor load	[53]
52	3	PRESS	2000/35	Lipid peaks can be used to distinguish poor prognostic HPV genotypes	[54]
10	7	STEAM and CSI	1400/36–75 1400/10	Lipid ratios were higher as a function of tumor grade, however the result was not significant	[25]
16–18	1.5	PRESS	1600/140	tCho levels remain high for patients with recurrence after radiation treatment	[56]
17	3	PRESS	1500/28 and 1500/144	Lipids are significantly elevated in partial or no response group, and Choline is elevated in good responder group	[24]
16	1.5	PRESS	2000/135	No MRS changes. ADC values of the tumor increased at the mid-treatment time point	[57]
62	1.5	PRESS CSI	888/135	No MRS changes. ADC values of the tumor were significantly lower compared to normal tissue	[58]

## Abbreviations

ADC	Apparent diffusion coefficient
AI	Artificial Intelligence
AUC	Area under the curve
Cho	Free choline
CIN	Cervical intraepithelial neoplasia
DWI	Diffusion weighted imaging
FOV	Field of view
GBM	Glioblastoma multiforme
GPC	Glycerophosphocholine
GR	Good responder
HPV	Human papillomavirus
MAS	Magical angle spinning
MRI	Magnetic resonance imaging
MRS	Magnetic resonance spectroscopy
MRSI	Magnetic resonance spectroscopic imaging
NED	No evidence of disease
NR	Non-responder
PCh	Phosphocholine
PD	Progression of disease
PR	Partial responder
PRESS	Point-resolved spectroscopy
RF	Radiofrequency
SIB	Simultaneous integrated boost
SNR	Signal-to-noise ratio
STEAM	Stimulated echo acquisition mode
tCho	Total choline
TE	Echo time
TM	Mixing time
TR	Repetition time
